# Prevalence and Factors Associated with Working Equid Lameness in Low- and Middle-Income Countries: A Systematic Review and Meta-Analysis

**DOI:** 10.3390/ani12223100

**Published:** 2022-11-10

**Authors:** Mathilde S. Merridale-Punter, Anke K. Wiethoelter, Charles M. El-Hage, Peta L. Hitchens

**Affiliations:** 1 Equine Lameness and Imaging Centre, Melbourne Veterinary School, University of Melbourne, 250 Princes Hwy, Werribee, VIC 3030, Australia; 2 Melbourne Veterinary School, Faculty of Veterinary and Agricultural Sciences, University of Melbourne, Parkville, VIC 3010, Australia

**Keywords:** working equids, lameness, gait abnormality, systematic review

## Abstract

**Simple Summary:**

In several low- and middle-income countries, equids support local communities by performing a variety of work, from transport to agriculture and other household duties. Issues such as lameness can therefore have significant implications, not only to the welfare of the animals, but also to the wellbeing of whole communities. However, the available evidence on working equid lameness is limited and highly varied, making its interpretation challenging. Therefore, we conducted a review of studies investigating lameness in working equids and analysed the combined findings from different studies. Furthermore, we looked at the main commonalities and differences in the current body of literature and made recommendations for standardization of methods and terminology when conducting research in this field. We found that over one third of working equids are reported to have lameness-related issues and that older, thinner animals, as well as those working every day of the week, are more likely to be lame. The results from this study could be used towards the improvement and tailoring of health and welfare programmes for working equids.

**Abstract:**

Lameness is an important concern in working equids of low- and middle-income communities (LMICs) with significant One Welfare implications. This study aims to determine the prevalence and influencing factors of lameness in working equids of LMICs. A systematic review and meta-analysis were performed to investigate pooled outcome prevalence using a random intercept regression model. Subgroup and sensitivity analysis were performed through meta-regression. A meta-analysis of study factors for lameness prevalence was performed. Sixty-four studies were included in the review. The pooled prevalence of lameness was 29.9% (*n* = 42, 95% CI 17–47%), while the pooled prevalence of gait abnormality was 62.9% (*n* = 12; 95% CI 31–87%). When considering both outcomes together, the pooled prevalence was 38.4% (*n* = 46; 95% CI 23–57%) with a significant (*p* = 0.02) difference between lameness (29.5%; 95% CI 16–48%) and gait abnormality (78.8%; 95% CI 40–95%). Species, country income level, gait assessed, and risk of bias did not significantly affect the pooled prevalence. Lower body condition scores, unresponsive attitudes, and old age were the most frequently reported factors positively associated with lameness-related outcomes. Working 7 days per week was positively associated with lameness. The standardization of outcome terminology, grading systems, and study factor categorization is recommended to enable more accurate interpretation and comparison between studies.

## 1. Introduction

It is estimated there are over 100 million working equids globally [1]. Horses, donkeys, and mules often perform essential roles in terms of employment, logistics, and domestic duties in many of the communities they serve [2,3], particularly in low and middle-income countries [4,5]. As such, any compromise to the health or welfare of these animals is also a threat to the livelihood of dependent communities, and may carry significant One Health implications [2,6].

While lameness is one of the most common health and welfare concerns in sport and pleasure horse populations [7,8,9], with significant implications for performance and survival [10], it is also a major issue in working equids in low- and middle-income countries [11,12,13,14,15,16]. Various factors associated with the presence or severity of lameness and gait abnormalities in working equids have been identified. These include low body condition score (BCS), [12,17], poor conformation [12], hoof and limb abnormalities, presence of skin lesions [14], poor cart condition, and driver inexperience [16]. However, the prevalence of reported lameness is highly variable across studied populations, ranging from 3% in Ethiopia [18] to up to 100% in India and Pakistan [11,12], albeit with marked differences in study sampling and methodology. Such heterogeneity of populations, methodology, and findings makes standardisation and interpretation of the scale and nature of lameness in working equids challenging, in addition to the difficulty of formulating effective preventative strategies.

We therefore undertook a systematic review of the available literature to (1) determine the prevalence of lameness-related outcomes across different populations of working equids in low- and middle-income countries (LMICs), (2) understand how the prevalence of lameness-related outcomes varies according to subgroups of interest such as equid species or country income level, and (3) identify key factors associated with lameness-related outcomes in working equids. Additionally, we aimed to identify gaps in the current knowledge of the epidemiology of lameness in working equids and recommend standardised reporting on this topic. Identifying common risk factors that may be generalisable across many working equid populations could be used towards the improvement and tailoring of health and welfare programmes for working equids.

## 2. Materials and Methods

### 2.1. Search Strategy

A review protocol was agreed to among coauthors (unregistered). The literature search was conducted in two steps by one reviewer (MSMP) on 26 July 2021. Firstly, they searched four electronic databases: CAB Abstracts; Scopus; Web of Science (Core Collections) and MEDLINE (Ovid). To maximise sensitivity, a three-term search using the Boolean operators AND and OR was conducted. The following search string was used, with format adaptations undergone to meet individual database requirements:

(1) (equid* OR horse* OR equine OR donkey* OR pony OR ponies OR mule OR mules)
  AND
  (working OR cart OR draft OR draught OR traction OR carriage OR caleche OR “horse drawn” OR cidomo)
  AND
  (lameness OR “gait abnormalit*” OR “gait irregular*” OR orthop* OR musculoskeletal OR injur* OR welfare)

Secondly, they searched Google Scholar to identify articles otherwise not detected in the specialised databases. Given the wide range of results and publication types retrieved by Google Scholar, only the first 100 results were considered. Due to the limitations of search word numbers on Google Scholar, a simplified search string was used:

(2) (equid* OR horse* OR equine OR donkey* OR pony OR ponies OR mule OR mules) (working OR cart OR draft OR draught OR traction OR carriage OR caleche OR “horse drawn” OR cidomo) (lameness)

The sensitivity of the search strings was assessed by comparing the results of a preliminary search to a benchmark of 13 publications known to be relevant to the review. No publication date limitations were imposed on the search. Title screening of the reference lists of included articles was also conducted to identify additional studies.

### 2.2. Article Screening and Eligibility Criteria

Retrieved studies were selected in two stages: stage 1 (title and abstract screening) and stage 2 (full-text screening of selected abstracts). For stage 1, eligibility criteria consisted of:

1. The topic of interest met;
2. Available in full text;
3. Original research; and
4. Available in English, French, Spanish, or Portuguese.

For stage 2, eligibility criteria consisted of:

1. Study meets eligible populations: working horses and/or mules and/or donkeys and/or ponies (equids used for work rather than sport or leisure activities and as draught animals for a form of transport or traction [19]) of low- and middle-income countries (LMICs) worldwide [20];
2. Study meets one or more of the outcomes of interest (prevalence and/or incidence and/or factors associated with lameness, gait abnormality, or gait irregularity); and
3. Primary empirical and secondary research, including cohort, case-control, cross-sectional studies, other observational studies, and retrospective data reviews.

During stage 1 screening, studies fulfilling all four eligibility criteria were moved to stage 2, full-text screening. Studies where the fulfilment of one or more eligibility criteria was unclear were also moved to stage 2 screening. Wherever the inclusion or exclusion criteria or data extraction and classification of an article were not clear, the article was assessed independently by at least two reviewers (AW, PLH).

### 2.3. Data Management and Extraction

EndNote20 (2013, Clarivate, Philadelphia, PA, USA [21]) was used to store and manage the retrieved search results. The references were then imported into the Covidence systematic review software (Veritas Health Innovation, available at www.covidence.org) [22], which was used for title and abstract screening as well as review management. Duplicates were identified and removed by the software (as well as manually) when the search results were evaluated.

Full-text studies that passed stages 1 and 2 of screening progressed to data extraction. Extracted data were compiled in Microsoft^®^ Excel^®^ (2011) (Redmond, WA, USA) and included: bibliography details, study type, study location, sample frame, study population, study methods, study outcomes (prevalence of lameness, gait abnormality, and/or lameness scores), outcome definition, outcome scale, gait assessed, and study factors for the outcomes of interest (positively associated/negatively associated/not significant). Studies were included for quantitative synthesis if they provided data on population size, cases of lameness or gait abnormality, prevalence of lameness or gait abnormality, and/or study factors for lameness or gait abnormality outcomes (study factor, sample size, number of cases, or direction of effect). The outcome of interest was defined as any asymmetry or deviation from soundness at walk or trot, irrespective of the lameness scale used. The prevalence of lameness-related outcomes was considered as the number of lameness or gait abnormality cases (numerator) over the number of animals assessed cross-sectionally for lameness or gait abnormality (denominator). Due to the lack of a clear and consistent definition in the literature for the threshold after which a gait abnormality or asymmetry is considered lameness on the visual gait assessment, both outcomes (lameness and gait abnormalities) were considered for analysis both together and as separate subgroups.

Countries were classified according to the World Bank’s income classification at the time data collection took place [20]. Where the income classification for the country changed during the data collection years of the study (*n* = 2 studies [23,24], *n* = 2 countries [India 2004–2007; Nepal 2018–2019], the country classification present for the first year of the study was selected. Where studies reported on combined outcomes from multiple countries with different income level classifications [17,24], the classification of the majority of countries in the study was considered for pooled analysis, and the analysis was performed both including and excluding these studies. Where data were incomplete or missing, typographical errors were present, or the raw data did not match the reported prevalence, the raw data, where available, were used in pooled analysis.

### 2.4. Study Validity and Risk of Bias Assessment

For each individual study, the risk of bias for external and internal validity in relation to the lameness outcomes reported was assessed using a modified version of the Hoy, Brooks et al. (2012) [25] checklist (Spreadsheet S1). Each study was assigned an internal and external validity score as well as an overall risk of bias based on the ability to fulfil each item on the checklist.

### 2.5. Statistical Analysis

Statistical analysis was performed using R software 4.2.1 (R Core Team, Vienna, Austria 2021) [26].

A meta-analysis of proportions was conducted for the overall and subgroup prevalence of lameness-related outcomes. Pooled prevalence estimates and overall summary proportion were calculated using the ‘metaprop’ function of the meta package in R [27]. A random effects model was used to calculate summary proportions due to the observed high study variability [28]. The maximum-likelihood method within the ‘metaprop’ function was used to estimate the study variance of effect size distribution (t^2^) [29]. Studies were weighted by subgroup (outcome assessed, country income level, equid species, gait assessed, and study risk of bias), and displayed as a forest plot with 95% confidence intervals (95% CI) [28]. Heterogeneity across studies was assessed within and between subgroups using the I^2^ statistic [30]. For studies reporting on more than one type of lameness-related outcome, a generalised linear mixed model (GLMM; family binomial; link logit) was generated to investigate whether there was a difference in the proportion of cases between the two outcomes (lameness and gait abnormality) in the same study. The number of cases over controls was the model outcome, while the fixed variable was the binary categorical type of lameness-related outcome (lameness or gait abnormality), with the research study functioning as a random effect, allowing the effect of the outcome to vary between studies. Where studies reported only on the prevalence of individual lameness grades but it was not clear from the scale used which grade(s) constituted lameness [31], those studies were not included in the pooled prevalence analysis.

Sensitivity analysis was performed in several ways. Pooled prevalence analysis was conducted both with and without studies classified as having a high risk of bias. Potential outliers and influential cases were identified using a Baujat plot [32] and a studentised residual test [33]. Observations with an absolute *z* value > 2 were investigated as potential outliers and pooled prevalence analysis was conducted both with and without the potential high-influence outliers. Further sensitivity analyses were conducted using meta-regression for all subgroup analysis [28].

Study factors reported for lameness-related outcomes were summarised together with their direction of association. For each study factor, the number of outcome positive and outcome negative cases was extracted, as well as its Odds Ratio (OR) and 95% confidence interval (95% CI). Where these data were not provided, the significance and direction of study factors was summarised according to the results reported or calculated via univariable analysis based on available data. Bonferroni correction for multiple comparisons was applied where appropriate, and the level of statistical significance was set at *p* < 0.05. A meta-analysis of study factors for the lameness-related outcome prevalence was also performed where possible, with the inclusion criteria being two or more studies reporting identical factor categories, regardless of statistical significance or sample size. Study factor meta-analysis was conducted for BCS, age (categorical), sex, equid species, hours worked per day, days worked per week, and work type. For one study where the full raw dataset was available [34], categories were created based on those from other studies in the meta-analysis to enable inclusion. However, if the relationship between study factors and the prevalence of lameness-related outcomes had not been investigated by the study [34], those studies were not included in the review summary of reported study factors. As BCS was reported qualitatively and quantitatively by different studies, a common categorisation was created through consensus of coauthors to enable analysis (BCS reported as 0–2/5, “thin” or “low” were categorised as “Poor”; 3/5 or “good” as “Ideal”; and 4–5/5 or “high” were categorised as “Fat”).

This review was conducted in accordance to the meta-analysis and systematic reviews of observational studies (MOOSE) guidelines [35] and is reported in line with the preferred reporting items for systematic reviews and meta-analysis (PRISMA) guidelines [36]. This review has not been registered.

## 3. Results

### 3.1. Systematic Review of Studies Investigating Working Equid Lameness in LMICs

**Figure 1:**
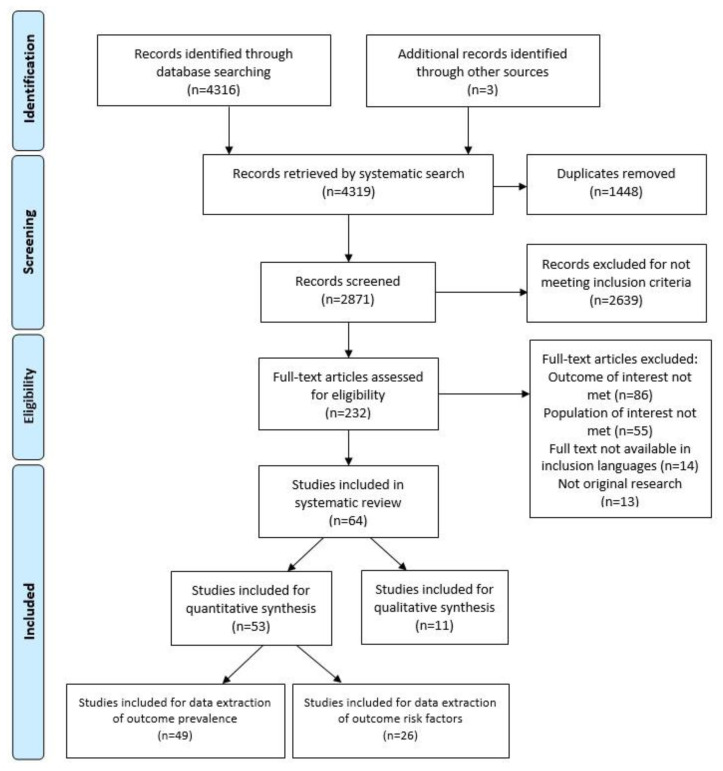
Preferred reporting items for systematic reviews and meta-analyses (PRISMA) flow diagram of studies identified and included for qualitative and quantitative synthesis of studies investigating working equid lameness in low- and middle-income countries, published from 2003 to 2021.

Studies identified through stage 1 and stage 2 of screening are summarised in Figure 1. Sixty-four studies were included in the systematic review, fifty-three studies (53/64; 82.8%) were included in quantitative analysis, and eleven studies (11/64; 17.2%) were included in qualitative synthesis (Figure 1). All studies retained for quantitative analysis were cross-sectional (53/53). Prevalence data were extracted from 92.5% (49/53) and study factors were extracted from 49.1% (26/53) of quantitative studies (Spreadsheet S2).

Most studies were published in English (61/64; 95.3%), with three (3/64; 4.7%) being published in Portuguese. The majority of eligible studies was published in scientific journals (54/64; 84.4%) while seven (7/64; 10.9%) were published in conference proceedings and three (3/64; 4.7%) were published in other types of scientific publications such as University journals.

**Figure 2:**
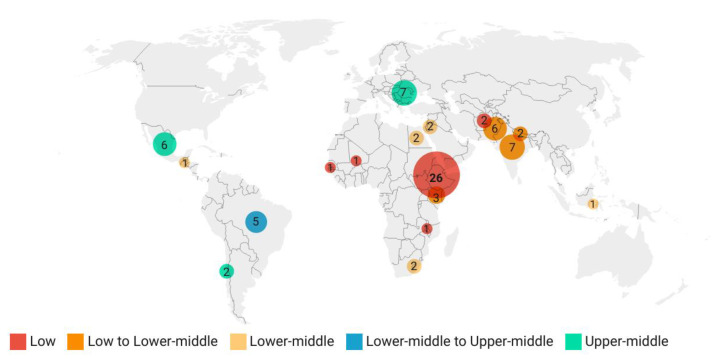
Target countries included in a systematic review of studies investigating working equid lameness in low- to middle-income countries, according to country income level at the time of data collection (bubble colour) and number of publications researching the country (bubble size and annotated number). Larger bubbles represent a higher number of publications. Where country income level classification was different for studies collecting data in different years, the income level was labelled as ‘Low to Lower-middle’ or ‘Lower-middle to Upper-middle’ accordingly. Figure created in Datawrapper [37].

The included studies were published between 2003 and 2021 and reported on data collected between 1997 and 2020. Ten studies (10/64; 15.6%) did not report study year(s). Most studies reported findings from an individual country (61/64; 95.3%), while others reported data from two (1/64; 1.6%), five (1/64; 1.6%), and nine (1/64; 1.6%) different countries (Spreadsheet S1). A total of 18 countries with World Bank income level classifications at the time of data collection ranging from low to upper-middle income were included (Figure 2).

### 3.2. Study Populations

Twenty-four studies (24/64; 37.5%) reported on donkey populations, followed by horses (21/64; 32.8%), mules (2/64; 3.1%), and ponies (1/64; 1.6%). Several studies reported mixed populations of equids such as horses and donkeys (2/64; 3.1%), horses, donkeys, and mules (13/64; 20.3%), and horses, donkeys, mules, and ponies (1/64; 1.6%) (Spreadsheet S1).

In 45.3% of studies (29/64), the sampled population included animals performing a variety of work types, such as agriculture, cart or pack transportation, agroforestry, and ceremonial work. Some studies reported exclusively on animals performing cart (15/64; 23.4%), pack (3/64; 4.7%), police (2/64; 3.1%), draught (3/64; 4.7%), and brick-kiln (3/64; 4.7%) work (conveying bricks at different stages of production [38]). The type of work conducted by the sampled equid population was not specified in nine (9/64; 14.1%) studies (Spreadsheet S1).

### 3.3. Study Focus and Study Arms

Of all of the studies included in the systematic review, 28.1% (18/64) reported on lameness-related or other orthopaedic outcomes as the primary focus of the research (Spreadsheet S1). Forty-one studies (41/64; 64.1%) reported on general health and welfare outcomes as the primary study focus, and five studies (5/64; 7.8%) had other primary objectives. Of studies included for quantitative analysis, those reporting lameness-related or orthopaedic outcomes as the primary focus represented 24.5% (13/53), while 66% (35/53) had general health and welfare or other outcomes (5/53; 9.4%) as the primary focus (Spreadsheet S1). The overall risk of bias for lameness-related outcomes was considered low in 14% of studies (9/64), moderate in 42.2% (27/64) of studies, and high in 43.6% (28/64) of studies (Spreadsheet S1).

Most studies included in quantitative analysis reported the prevalence of lameness and/or gait abnormality (26/53; 49.1%), or both prevalence and study factors (23/53; 43.4%). One study (1/53; 1.9%) reported the prevalence of severe lameness only, one study (1/53; 1.9%) reported only study factors for abnormal gait, and two studies (2/53; 3.8%) reported mean or prevalence of lameness grades and study factors (Spreadsheet S2).

Study arms were defined as subsets of data extracted from the same study, based on subgroups of the population (by equid species or country) or by reported outcome. Forty studies (40/53; 75.5%) were treated as only one arm for the overall study, while multiple study arms were extracted from the remaining 13 studies (13/53; 24.5%). Six (6/53; 11.3%) studies provided discrete equid species arms, four (4/53; 7.5%) studies provided different outcome arms, and three (3/53; 5.7%) studies provided separate country or environment arms. Overall, data were extracted as 91 separate study arms from 53 studies (Spreadsheet S2).

### 3.4. Detection and Definition of Lameness Outcomes

The outcomes of interest included for quantitative analysis were lameness (40/53; 75.5%) and gait abnormality (9/53; 16.98%). Four studies (4/53; 7.5%) reported on both outcomes.

Most studies (34/53; 64.2%) did not specify the gait at which lameness or gait abnormality was assessed. In 28.3% of studies (15/53), the outcome was assessed at the walk, and in 7.5% of studies (4/53), the outcome was assessed at both walk and trot. Multiple grading scales for the assessment of outcome severity were used. Twenty-one studies (21/53; 39.6%) did not specify how the outcome classification was achieved. The most commonly used grading system was the American Association of Equine Practitioners (AAEP) lameness scale (6/53; 11.3%) [39] (Table 1).

### 3.5. Prevalence of Lameness-Related Outcomes

**Figure 3:**
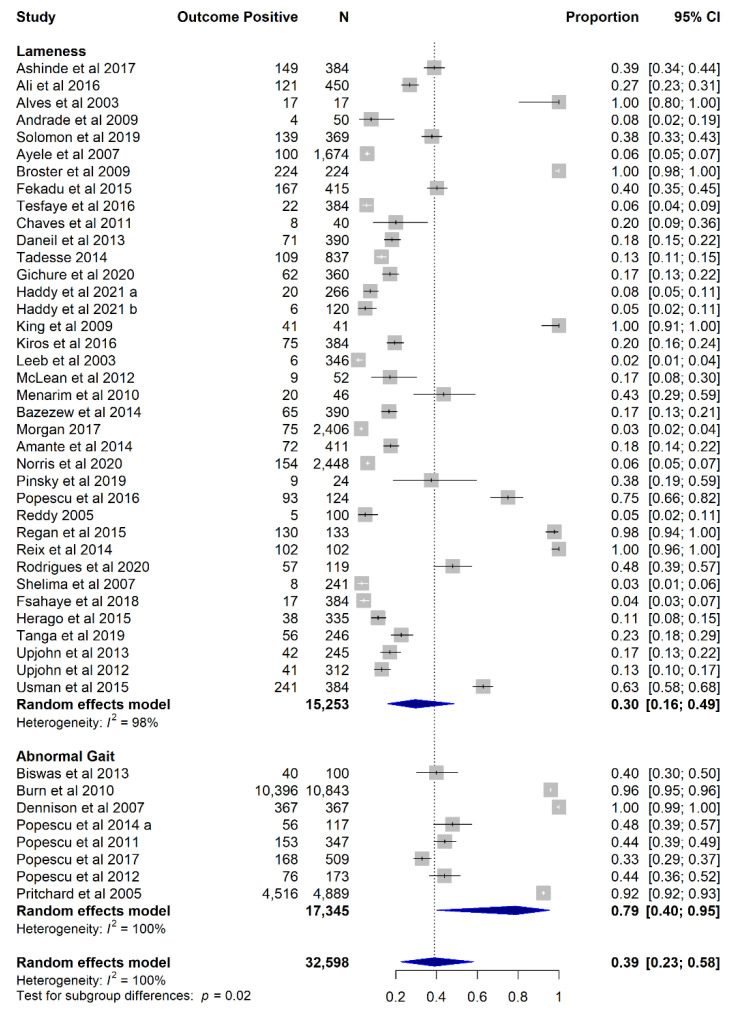
Forest plot illustrating the pooled prevalence of all lameness-related outcomes (lameness and gait abnormality) considered together and as outcome subgroups in a meta-analysis of proportions of studies investigating working equid lameness in low- and middle-income countries, 2003 to 2021: Ashinde et al. 2017 [44], Ali et al. 2016 [14], Alves et al. 2003 [45], Andrade et al. 2009 [46], Solomon et al. 2019 [47], Ayele et al. 2007 [48], Broster et al. 2009 [11], Fekadu et al. 2015 [49], Tesfaye et al. 2016 [50], Chaves et al. 2011 [51], Daneil et al. 2013 [52], Tadesse 2014 [53], Gichure et al. 2020 [54], Haddy et al. 2021a [55], Haddy et al. 2021b [34], King et al. 2009 [13], Kiros et al. 2016 [15], Leeb et al. 2003 [56], McLean et al. 2012 [57], Menarim et al. 2010 [58], Bazezew et al. 2014 [16], Morgan 2017 [18], Amante et al. 2014 [59], Norris et al. 2020 [23], Pinsky et al. 2019 [60], Popescu et al. 2016 [61], Reddy 2005 [62], Regan et al. 2015 [63], Reix et al. 2014 [12], Rodrigues et al. 2020 [64], Shelima et al. 2007 [65], Fsahaye et al. 2018 [66], Herago et al. 2015 [67], Tanga et al. 2019 [68], Upjohn et al. 2013 [69], Upjohn et al. 2012 [70], Usman et al. 2015 [71], Biswas et al. 2013 [72], Burn et al. 2010 [24], Dennison et al. 2007 [73], Popescu et al. 2014a [74], Popescu et al. 2011 [75], Popescu et al. 2017 [76], Popescu et al. 2012 [77], Pritchard et al. 2005 [17].

The pooled prevalence of lameness was 30% (*n* = 41; 95% CI 17–48%; t^2^ 5.69; I^2^ 98.5%), while the pooled prevalence of gait abnormality was 62.9% (*n* = 12; 95% CI 31–87%; t^2^ 5.49; I^2^ 99.7%). When considering all lameness-related outcomes (lameness and gait abnormality) together and excluding studies reporting on both outcomes (*n* = 4), the pooled prevalence was 38.9% (*n* = 45; 95% CI 23–58%; t^2^ 6.85; I^2^ 99.6%), with the pooled prevalence of lameness (29.8%; 95% CI 16–49%) being lower than that of gait abnormality (79%; 95% CI 40–95%; *p* = 0.025) (Figure 3).

The removal of the study with highest influence on the overall result [24] decreased the pooled prevalence of lameness-related outcomes to 37% (95% CI 21–56%), rendering the difference between lameness and gait abnormality prevalence not significant (*p* = 0.068; Table S1). However, the high influence of this study was attributed to its large sample size (*n* = 10,843), thus the authors found no justifiable reason to exclude the study as an outlier from subsequent analysis. For studies reporting on both lameness and gait abnormality outcomes (*n* = 4), there was no difference between the pooled prevalences of lameness (36%; 95% CI 9–75%; t^2^ 3.02; I^2^ 99%) and gait abnormality (27%; 95% CI 9–60%; t^2^ 1.96; I^2^ 99%) (OR 1.56; 95% CI 0.92–2.6; *p* = 0.098; Figures S1–S5).

The prevalence of lameness-related outcomes did not differ according to country income level, equid species, gait assessed (walk/trot), and risk of bias subgroups (*p* > 0.05, Table S1 and Figures S1–S5). Sensitivity analyses did not affect the significance of subgroup assessments (Table S1).

### 3.6. Study Factors for Lameness-Related Outcomes

**Figure 4:**
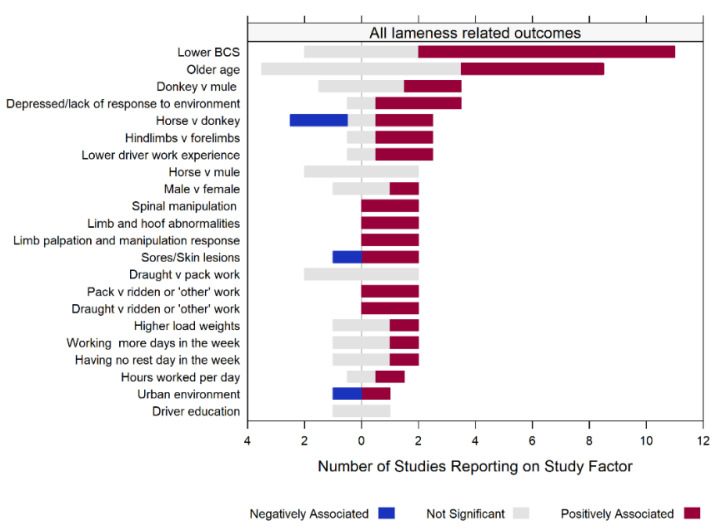
Summary of study factors for all lameness-related outcomes (prevalence of lameness, prevalence of gait abnormality, or lameness scores combined) reported by more than one study in a systematic review of studies investigating working equid lameness in low- and middle-income countries ranging from 2003 to 2021. For binary study factors, the reference category is listed last. The direction of associations reported is represented by different colours, with positively associated factors being those associated with a higher prevalence or severity of a lameness-related outcome, and negatively associated factors being those related to a lower outcome prevalence or grade. Each study contributes only once to each factor as the overall study population. Significance levels and directions of study factors for different study arms are not represented in this figure and may differ from that extracted for the overall study population.

The study factors for lameness-related outcomes reported by the included studies as well as the direction of association reported or calculated based on available data are summarised in Supplementary File S5 (Spreadsheet S3) [78]. In total, 60 factors were extracted for the prevalence of lameness, 25 factors were extracted for the prevalence of gait abnormality, and 36 factors were extracted for higher lameness scores from 26 studies and 44 study arms. Study factors reported by more than one study for all lameness-related outcomes combined are presented in Figure 4. Low body condition score, equid age, equid sex, equid species, equid demeanour, limb score, driver experience, physical assessment parameters (e.g., hoof angle, tendon enlargement, or pain on palpation), type of work, cart load weight, working patterns, and environment were investigated in more than one study and were considered a factor positively associated with lameness-related outcomes by at least one study, while the presence of skin lesions, urban environment, and horses in relation to donkeys has been considered by at least one study to be associated with lower or less severe lameness-related outcomes (Figure 4). Body condition score was the most frequently investigated study factor, reported as being associated with a higher prevalence or severity of lameness-related outcomes in nine studies and as nonsignificant in four studies. Age was investigated in 12 studies and older age was positively associated with lameness-related outcomes in 5 studies. No studies found driver education to be associated with lameness-related outcomes, nor were there differences in prevalence of lameness-related outcomes between horses and mules. No differences in prevalence of lameness-related outcomes were found between draught and pack type of work at the overall study population level (Figure 4), although draught has been reported as a positively associated factor in donkeys at the species study arm level [79] (Spreadsheet S3).

### 3.7. Meta-Analysis of Study Factors

**Figure 5:**
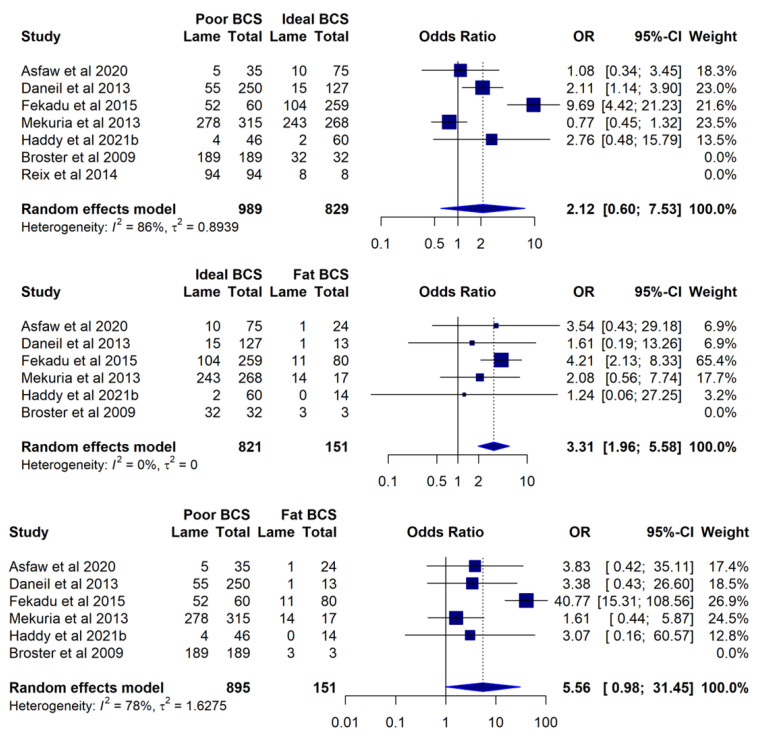
A meta-analysis of studies investigating working equid lameness in low- and middle-income countries from 2003 to 2021, with available data for the prevalence of lameness per body condition score (BCS) category: Asfaw et al. 2020 [80], Daneil et al. 2013 [52], Fekadu et al. 2015 [49], Mekuria et al. 2013 [79], Haddy et al. 2021b [34], Broster et al. 2009 [11], Reix et al. 2014 [12]. Reference categories are listed on the right.

Despite lower BCSs being reported as a factor associated with a higher prevalence or severity of lameness-related outcomes in 69.2% (9/13) of studies investigating this factor, where data for prevalence of lameness per BCS category were available (*n* = 7 studies), a meta-analysis of the pooled lameness prevalence according to the BCS category showed that only animals with an ideal BCS (score 3/5) were significantly more likely to be lame in relation to fat (scores 4–5/5) animals (OR 3.31; 95% CI 1.96–5.58) (Figure 5).

**Figure 6:**
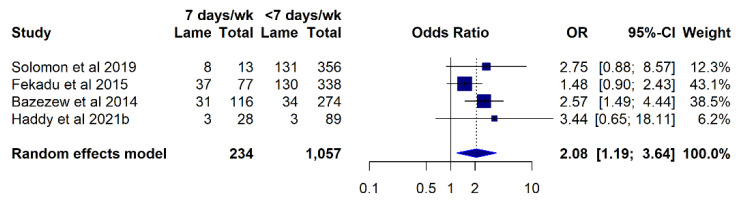
A meta-analysis of studies investigating working equid lameness in low- and middle-income countries ranging from 2003 to 2021, with available data for the prevalence of lameness according to number of days worked per week: Solomon et al. 2019 [47], Fekadu et al. 2015 [49], Bazezew et al. 2014 [16], Haddy et al. 2021b [34]. The reference category is listed on the right.

Equids working seven days per week were more likely to be lame compared to equids working fewer days per week (OR 2.08; 95% CI 1.19–3.64) (Figure 6). The remaining meta-analysis with the available data showed no other significant study factors for the prevalence of lameness-related outcomes.

### 3.8. Studies Included for Qualitative Assessment Only

Seventeen percent of studies (11/64) investigated lameness-related outcomes in working equids but were included in qualitative synthesis [81,82,83,84,85,86,87,88,89,90] (Spreadsheet S4). These studies were not eligible for data extraction, as the sampled population had other concurrent abnormalities (3/11; 27.3%), investigated populations of exclusively lame animals (2/11; 18.2%), the outcome was unclear or not strictly lameness-related (2/11; 18.2%), or did not provide quantitative data for the extraction or calculation of the outcomes of interest (4/11; 36.4%). Most of these ineligible studies (8/11; 72.7%) reported on factors associated with lameness-related outcomes, while others described the proportion of various lameness-related outcomes (5/11; 45.5%) or described and characterised lameness in the sampled population (3/11; 27.3%) (Spreadsheet S4). Weak positive correlations were found between the length of stable scratch marks and a greater incidence of lameness [86], and weak negative correlations were found between abnormal behaviours and rate of lameness in police working horses [87]. Hoof moulding and hardware was reported to be associated with lameness [91], while animals transporting people by cart and a BCS either below or above ideal were associated with higher lameness scores [84]. Equid age, BCS, sex [88], or species [82,88] were not significantly associated with lameness-related outcomes.

## 4. Discussion

This review has compiled important aspects of the epidemiology of lameness in working equids and identified areas for improvement in the reporting of lameness-related outcomes in this group of animals. The overall pooled prevalence of lameness-related outcomes suggests that more than a third of working equids in low- and middle-income communities are lame or have gait abnormalities at a given time. However, the high I^2^ values obtained when calculating pooled prevalence estimates indicate high study heterogeneity, with marked differences in study sample size. The high variation in prevalence across studies may be due to the observational and cross-sectional nature of the studies included, as well as the specific equid population sampled and other context-dependent differences. As such, the interpretation of pooled prevalence estimates in such a heterogeneous body of literature must be done with caution. Additionally, not all publications were published in peer-reviewed scientific journals, and most studies were considered to have a moderate to high degree of bias in relation to lameness-related outcomes.

The prevalence of lameness in included studies ranged from 3% [18,65] to 100% [11,12,13,45], while the prevalence of gait abnormalities had a narrower range but a significantly higher pooled prevalence than that of lameness. However, in studies that reported both outcomes simultaneously [79,80,92,93] the pooled prevalence of lameness was marginally higher than that of gait abnormalities. This highlights the lack of clarity regarding the differences in definition of lameness and other gait abnormalities in the literature. Whereas gait abnormalities might include neurological, conformational, or postural asymmetries of gait, differentiation between these and mechanical or pain-induced lameness is often subjective, particularly when visually appraising subclinical lameness [94]. Nevertheless, if interpreting both outcomes in conjunction, a high proportion of animals appear to have locomotor disorders, while if considering them separately, a pooled prevalence of gait abnormalities of nearly 80% raises additional and important questions as to other locomotor or neurological disfunctions potentially compromising welfare in working equids. The gait at which lameness-related outcomes were assessed was also inconsistent between reports and was often not specified. Although subgroup analysis did not show the pooled outcome prevalence to be affected by study risk of bias or gait assessed, this might nonetheless influence diagnostic sensitivity and choice of outcome terminology. Several studies also did not specify the lameness scale, appraiser, or surface conditions under which the assessment was conducted, which may impact the accuracy of lameness detection [95,96,97]. The wide range of lameness scales and grading systems used further contributes to the heterogeneity of the literature and makes a comparison of severity difficult. Standardising the way in which lameness-related outcomes are assessed and reported would therefore benefit the general understanding of their epidemiology and allow for better comparison and interpretation of the literature, as well as identification of common risk factors and potential solutions.

Equids working seven days per week were more likely to be lame than those working fewer days. Working the animals every day is common practice in parts of the world [14,16,34] and is often a reflection of the intense socioeconomic pressures faced by their users [2,5]. Still, providing at least one rest day a week would be an appropriate recommendation to reduce the risk of longer off-work periods due to lameness and the associated income losses, as well as to help safeguard animal welfare. A lower BCS was most frequently reported to be associated with a higher prevalence or severity of lameness [11,12,15,16,17,24,31,47,49]. However, the cause–effect direction of this association is not clear, and it could simply reflect a noncausal association with other poor welfare outcomes. This could be due to thin animals having a lower fat coverage and less protection from trauma and injury [17] potentially being more prone to lameness. Alternatively, a lower body condition could be a reflection of increased age, reduced ambulation and access to food, or higher energy expenditure due to lameness in the first place, as seen with other underlying diseases [98]. Lastly, it is also possible that poor welfare begets further welfare compromise, and animals with lower body condition scores may be reflective of poor management practices, including lack of attention to dietary requirements or potential causes of lameness, excess workload and energy expenditure, or failure to detect and manage lameness early. Conflictingly, meta-analysis of BCS as a study factor for the prevalence of lameness showed only animals with ideal BCS to be more likely to be lame in relation to fat animals, although this is likely influenced by the very low number of studies with available data and the low number of fat animals in the analysis. In addition, subjective differences in the classification of BCS by different studies could play a factor, as two studies [79,80] classified BCS 3/5 as moderate and 4–5/5 as good, which differs from what is generally considered an ideal and fat BCS, respectively [99]. Fekadu and colleagues [49] used a 5-point BCS scale but present their data in only three qualitative categories, which could explain the markedly different odds ratios obtained from this study. Older age was also frequently reported in positive association with lameness-related outcomes. Age is a known risk factor for lameness in other populations of nonworking equids [100,101,102], but the generally lower life expectancy of working equids [103,104] grants it additional welfare significance. Behavioural indicators such as depressed attitude, apathy, and lack of response to environment have also been reported by several authors in association with lameness-related outcomes. This could be related to stress or pain in direct association with lameness, but it also could be a manifestation of poor physical health [105], withdrawn attitude under unfavourable conditions [106], or older age and low BCS [105], which are themselves associated with lameness [17,24]. However, the inherent subjectivity between the various measures of unresponsiveness and differences in categorisation between different studies should be considered.

The pooled prevalence of lameness-related outcomes did not significantly vary according to equid species or country income level subgroups. In some populations of donkeys, risk factors for orthopaedic conditions are different to those for the horse [107]. Although several studies have reported equid species as a factor associated with lameness outcomes [17,24,79], no differences were identified in pooled analysis. The fact that no differences in pooled prevalence according to country income level were identified does not exclude the possibility that the economic capacity of the equid users influences lameness prevalence, since equid welfare status can be affected by socioeconomic pressures [2,38,108]. Differences may be evident at the community and individual levels.

Reports on the influence of specific work type on lameness-related outcomes varied. In the pooled prevalence analysis of the entire study sample, there was no difference between draught and pack work and the likelihood of having gait abnormalities, although both were individually more associated with lameness-related outcomes in relation to ridden or other types of work. However, at the species level from different study arms, draught work has been reported as a positively associated factor in relation to pack work in donkeys [79]. Nevertheless, the interpretation of this effect is limited, as these work type categorisations are broad and encompass several variations within the specific type of draught or pack work, the equipment used, working conditions, and work practices themselves, which can influence the outcome. Additionally, although the reaction to spinal pressure has only been investigated by two studies [11,12], both reported this as a factor associated with higher lameness scores, which should be accounted for when considering the equipment and loading of working equids. While studies investigating the effect of driver education on lameness prevalence found no significant relationship, lower driver work experience was reported as a factor positively associated with lameness in working donkeys [47] and mules [16] in Ethiopia, which is relevant to the demographic tailoring of veterinary and community advice. Equid species, skin lesions, and environment were the only factors investigated with opposing evidence as to the direction of the association with lameness outcomes. Namangale and colleagues [31] reported the presence of skin lesions to be negatively correlated with lameness score, but in contrast, higher wound scores were positively correlated with higher lameness scores. However, this was a study based on a sample of only 48 donkeys that was considered to have a high risk of bias, so findings should be interpreted in context. Rural environments were reported both in positive [24] and negative [18] association with the prevalence of gait abnormalities, which could be due to variations in the distribution of sampled species, countries, type of work, or cultural work practices. Interestingly, although not represented at the study level or in pooled subgroup analysis, at the study-arm level, donkeys are more frequently reported as being associated with lameness outcomes than horses or mules [17,23,24]. Socio-economic and cultural perceptions of donkeys, together with their generally lower economic value, might contribute to the overworking of these animals, potentially making them more prone to lameness. Nevertheless, this study discusses factors associated with higher or lower prevalence and severity of lameness-related outcomes in a sample of cross-sectional studies. As such, it is important to bear in mind that the causality of their relationship with lameness or gait abnormality cannot be inferred. Some factors could potentially be modified to reduce lameness outcomes, but others may simply reflect associations with other poor welfare outcomes in animals with multiple welfare issues. Further longitudinal studies would be needed to investigate modifiable risk factors for lameness-related outcomes in working equids.

The body of literature retrieved by this systematic review is highly heterogeneous in terms of target population, sampling frame and size, species sampled, work type, case definition, diagnostic methods, and variable categorisation, among other factors. However, this is the most comprehensive systematic review of working equid lameness and gait abnormalities to date and provides a valuable insight into the epidemiology of lameness-related outcomes and the main areas for improvement in reporting on this topic. Several studies were considered to have publication bias due to unclear internal or external validity [109]. Nevertheless, this represents only one component of the critical consideration of evidence [109], and the exclusion of studies with a high risk of bias did not significantly influence pooled outcome prevalence. The markedly high study heterogeneity makes direct comparisons of results inappropriate, and the summary of study factors reported must be viewed in context of the study’s sample size and the likelihood of statistical significance. However, sensitivity analysis was conducted in different ways and did not affect the significance of results. Although these findings should be interpreted cautiously and in context, the pooled prevalence of lameness-related outcomes and respective confidence intervals is nonetheless a valuable indicator of the magnitude of this welfare problem.

## 5. Conclusions

Lameness is an important issue affecting working equids. Animals that worked every day of the week were more likely to be lame, and the groups most frequently reported in association with lameness were older animals, those with lower BCSs, and those with dull or unresponsive attitudes. Although the prevalence of gait abnormalities was higher than that of lameness, these terms are frequently used interchangeably in the literature and, often, no clear outcome definition is provided. A standardisation of terminology, grading systems, and categorisation of study factors used in the investigation of lameness is therefore required. As working equid owners often fail to detect lameness in their animals [61], and may lack the access or capacity to seek veterinary care [2], understanding risk factors for lameness in working equids would allow for a prompter identification of populations and individuals at risk and for the tailoring of any veterinary and community engagement interventions accordingly. The presence of a rest day in the week, BCS, demeanour, and age appear to be useful noninvasive indicators when assessing the likelihood of lameness outcomes and should be incorporated into their prevention and monitoring strategies.

## Figures and Tables

**Table 1:** Grading scales of lameness or gait abnormality used for the assessment of outcome severity in a systematic review of studies (*n* = 53) investigating working equid lameness in low- and middle-income countries, 2003 to 2021.

Grading Scale Used	Number of Studies	
Unknown	21	39.6%
Normal/Abnormal	7	13.2%
1–5 AAEP lameness scale [40]	4	7.5%
Modified AAEP lameness scale	2	3.8%
1–5 Hands on donkey tool [41]	3	5.7%
1–5 (unspecified)	2	3.8%
EARS tool [42]	4	7.5%
Modified Busschers and Van Weeren (0–4) [43]	1	1.9%
0–10 (unspecified)	1	1.9%
0–2 (unspecified)	1	1.9%
0–3 (unspecified)	1	1.9%
0–5 (unspecified)	1	1.9%
Mild, severe	1	1.9%
Low, moderate, high	1	1.9%
Low, moderate, high, immobile	3	5.7%

## Data Availability

Data supporting the reported results are contained within the article or can be found in the Supplementary Materials and original research articles included in the review.

## Supplementary Materials

The following supporting information can be downloaded at: https://www.mdpi.com/article/10.3390/ani12223100/s1, Spreadsheet S1: All studies included for review; Spreadsheet S2: Extracted data from all studies and study arms included for quantitative analysis; Table S1: Sensitivity analysis for meta-analysis of lameness-related outcomes; Figures S1–S5: Forest plots for alternative subgroup meta-analysis of lameness-related outcomes; Spreadsheet S3: Summary of study factors reported against lameness-related outcomes; Spreadsheet S4: Studies included for qualitative comment. Ref. [110] was cited in Spreadsheet.
