# Supplementary material for: Prevalence and Factors Associated with Working Equid Lameness in Low- and Middle-Income Countries: A Systematic Review and Meta-Analysis
Source: Animals (Basel). 2022 Nov 10;12(22):3100. doi: 10.3390/ani12223100 (PMC9686919; doi:10.3390/ani12223100)

Forest plots for alternative subgroup analysis

Figure S1 – Forest plot illustrating the pooled prevalence and outcome subgroup comparison of studies reporting on both lameness and gait abnormality outcomes in a meta-analysis of proportions of studies investigating working equid lameness in low- and middle-income countries, 2013 to 2020: Asfaw et al 2020 [79], Mola et al 2017 [93], Niraj et al 2014 [92], Mekuria et al 2013 [80]. A generalised linear mixed model (GLMM) was generated to investigate correlations between two outcomes in the same study.

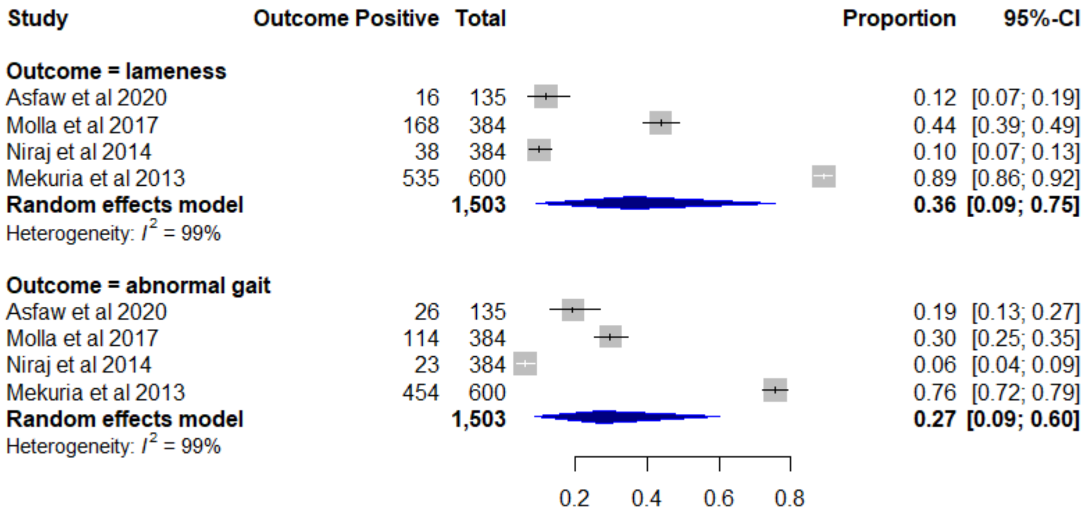

Figure S2 – Forest plot illustrating the pooled prevalence of all lameness-related outcomes (lameness and gait abnormality) according to country income-level subgroups in a meta-analysis of proportions of studies investigating working equid lameness in low- and middle-income countries, 2003 to 2021: Ashinde et al 2017 [44], Ali et al 2016 [14], Alves et al 2003 [45], Andrade et al 2009 [46], Solomon et al 2019 [47], Ayele et al 2007 [48], Broster et al 2009 [11], Fekadu et al 2015 [49], Tesfaye et al 2016 [50], Chaves et al 2011 [51], Daneil et al 2013 [52], Tadesse 2014 [53], Gichure et al 2020 [54], Haddy et al 2021a [55], Haddy et al 2021b [34], King et al 2009 [13], Kiros et al 2016 [15], Leeb et al 2003 [56], McLean et al 2012 [57], Menarim et al 2010 [58], Bazezew et al 2014 [16], Morgan 2017 [18], Amante et al 2014 [59], Norris et al 2020 [23], Pinsky et al 2019 [60], Popescu et al 2016 [61] , Reddy 2005 [62], Regan et al 2015 [63], Reix et al 2014 [12], Rodrigues et al 2020 [64], Shelima et al 2007 [65], Fsahaye et al 2018 [66], Herago et al 2015 [67], Tanga et al 2019 [68], Upjohn et al 2013 [69], Upjohn et al 2012 [70], Usman et al 2015 [71], Biswas et al 2013 [72], Burn et al 2010 [24], Dennison et al 2007 [73], Popescu et al 2014a [74], Popescu et al 2011 [75], Popescu et al 2017 [76], Popescu et al 2012 [77], Pritchard et al 2005 [17]. Countries were classified according to the World Bank's income classification at the time data collection took place [21].

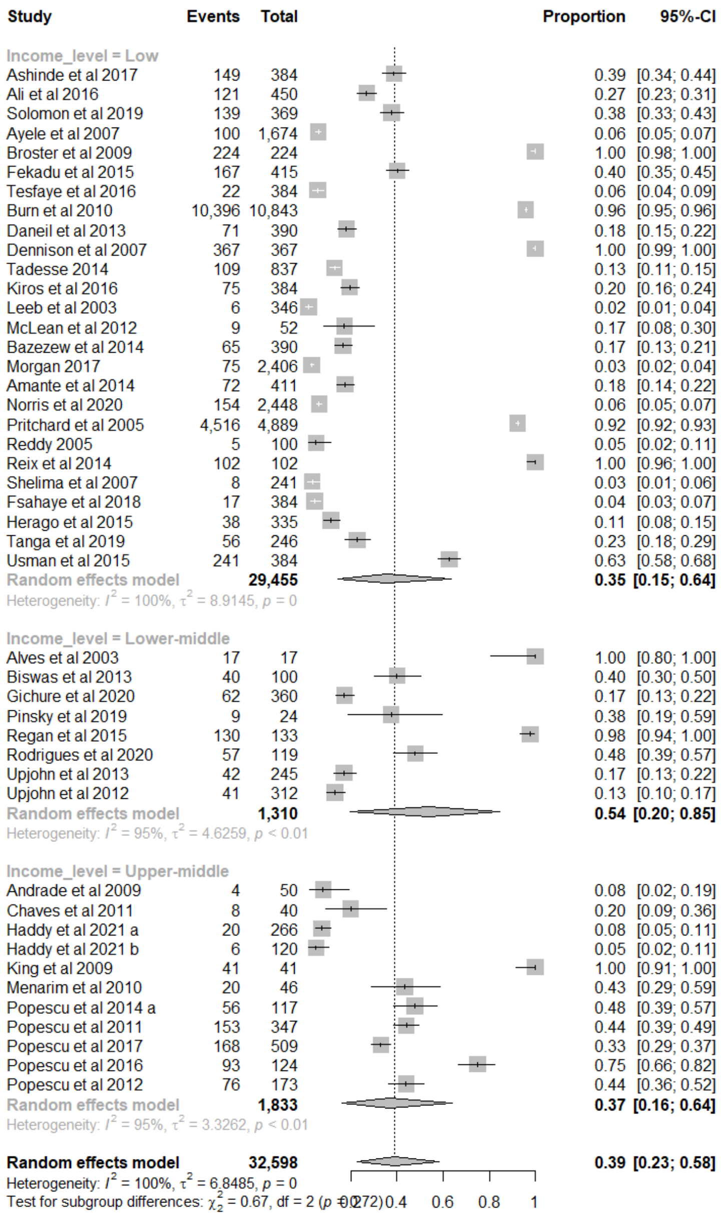

Figure S3 – Forest plot illustrating the pooled prevalence of all lameness-related outcomes (lameness and gait abnormality) according to equid species subgroups in a meta-analysis of proportions of studies investigating working equid lameness in low- and middle-income countries, 2003 to 2021: Ashinde et al 2017 [44], Ali et al 2016 [14], Alves et al 2003 [45], Andrade et al 2009 [46], Solomon et al 2019 [47], Ayele et al 2007 [48], Broster et al 2009 [11], Fekadu et al 2015 [49], Tesfaye et al 2016 [50], Chaves et al 2011 [51], Daneil et al 2013 [52], Tadesse 2014 [53], Gichure et al 2020 [54], Haddy et al 2021a [55], Haddy et al 2021b [34], King et al 2009 [13], Kiros et al 2016 [15], Leeb et al 2003 [56], McLean et al 2012 [57], Menarim et al 2010 [58], Bazezew et al 2014 [16], Morgan 2017 [18], Amante et al 2014 [59], Norris et al 2020 [23], Pinsky et al 2019 [60], Popescu et al 2016 [61] , Reddy 2005 [62], Regan et al 2015 [63], Reix et al 2014 [12], Rodrigues et al 2020 [64], Shelima et al 2007 [65], Fsahaye et al 2018 [66], Herago et al 2015 [67], Tanga et al 2019 [68], Upjohn et al 2013 [69], Upjohn et al 2012 [70], Usman et al 2015 [71], Biswas et al 2013 [72], Burn et al 2010 [24], Dennison et al 2007 [73], Popescu et al 2014a [74], Popescu et al 2011 [75], Popescu et al 2017 [76], Popescu et al 2012 [77], Pritchard et al 2005 [17].

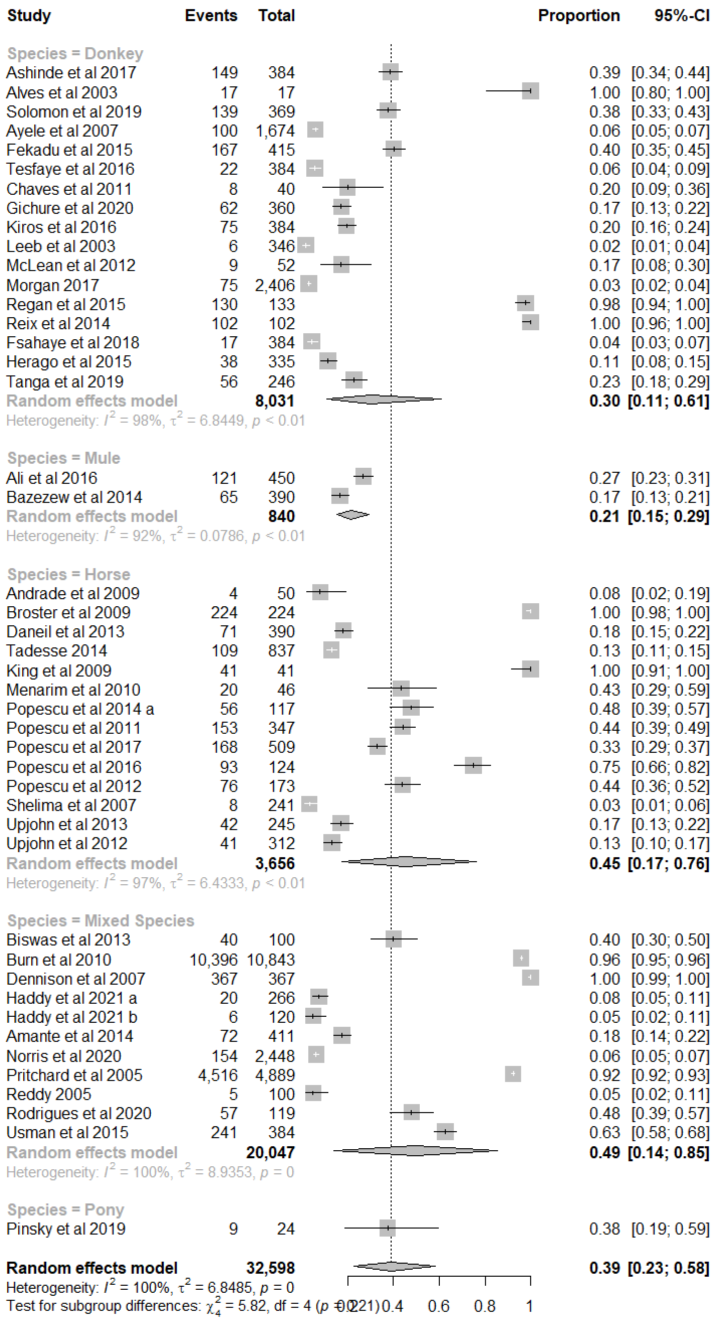

Figure S4 – Forest plot illustrating the pooled prevalence of all lameness-related outcomes (lameness and gait abnormality) according to gait assessed for lameness detection subgroups in a meta-analysis of proportions of studies investigating working equid lameness in low- and middle-income countries, 2003 to 2021: Ashinde et al 2017 [44], Ali et al 2016 [14], Alves et al 2003 [45], Andrade et al 2009 [46], Solomon et al 2019 [47], Ayele et al 2007 [48], Broster et al 2009 [11], Fekadu et al 2015 [49], Tesfaye et al 2016 [50], Chaves et al 2011 [51], Daneil et al 2013 [52], Tadesse 2014 [53], Gichure et al 2020 [54], Haddy et al 2021a [55], Haddy et al 2021b [34], King et al 2009 [13], Kiros et al 2016 [15], Leeb et al 2003 [56], McLean et al 2012 [57], Menarim et al 2010 [58], Bazezew et al 2014 [16], Morgan 2017 [18], Amante et al 2014 [59], Norris et al 2020 [23], Pinsky et al 2019 [60], Popescu et al 2016 [61] , Reddy 2005 [62], Regan et al 2015 [63], Reix et al 2014 [12], Rodrigues et al 2020 [64], Shelima et al 2007 [65], Fsahaye et al 2018 [66], Herago et al 2015 [67], Tanga et al 2019 [68], Upjohn et al 2013 [69], Upjohn et al 2012 [70], Usman et al 2015 [71], Biswas et al 2013 [72], Burn et al 2010 [24], Dennison et al 2007 [73], Popescu et al 2014a [74], Popescu et al 2011 [75], Popescu et al 2017 [76], Popescu et al 2012 [77], Pritchard et al 2005 [17].

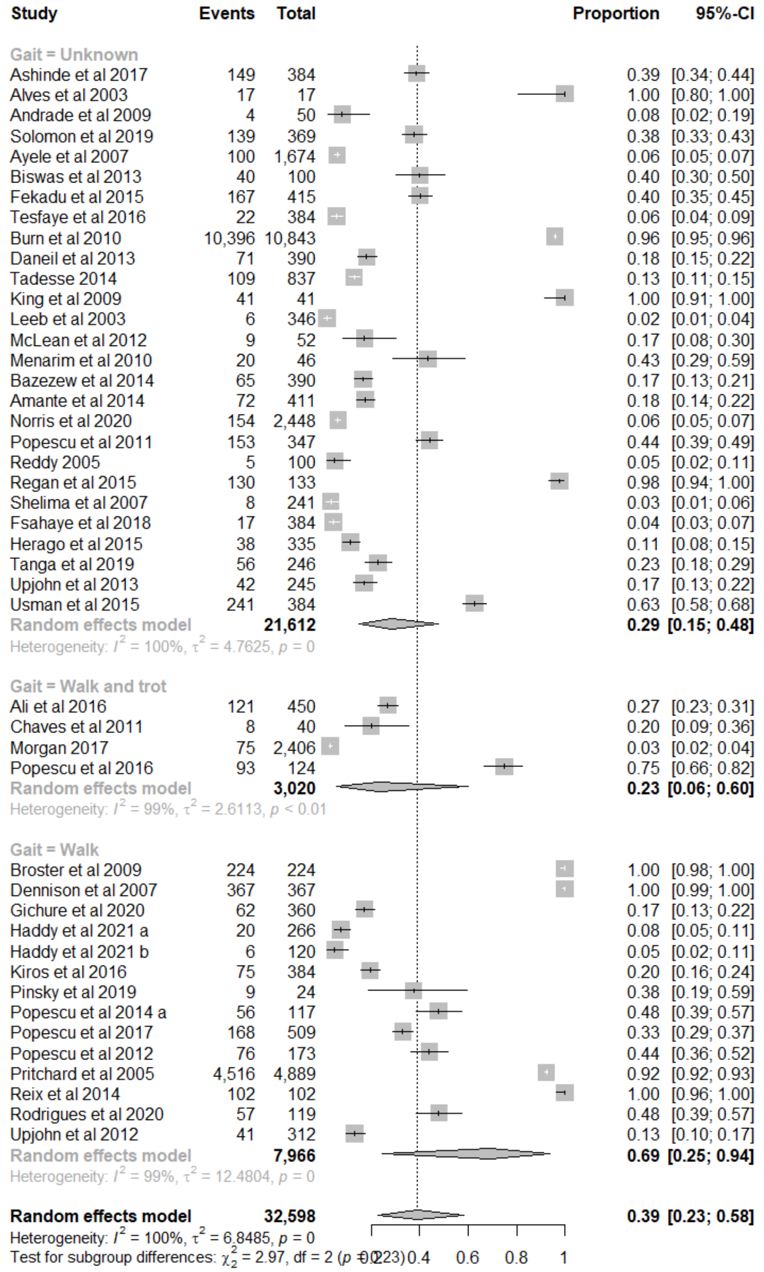

Figure S5 – Forest plot illustrating the pooled prevalence of all lameness-related outcomes (lameness and gait abnormality) according to study risk of bias subgroups in a meta-analysis of proportions of studies investigating working equid lameness in low- and middle-income countries, 2003 to 2021: Ashinde et al 2017 [44], Ali et al 2016 [14], Alves et al 2003 [45], Andrade et al 2009 [46], Solomon et al 2019 [47], Ayele et al 2007 [48], Broster et al 2009 [11], Fekadu et al 2015 [49], Tesfaye et al 2016 [50], Chaves et al 2011 [51], Daneil et al 2013 [52], Tadesse 2014 [53], Gichure et al 2020 [54], Haddy et al 2021a [55], Haddy et al 2021b [34], King et al 2009 [13], Kiros et al 2016 [15], Leeb et al 2003 [56], McLean et al 2012 [57], Menarim et al 2010 [58], Bazezew et al 2014 [16], Morgan 2017 [18], Amante et al 2014 [59], Norris et al 2020 [23], Pinsky et al 2019 [60], Popescu et al 2016 [61] , Reddy 2005 [62], Regan et al 2015 [63], Reix et al 2014 [12], Rodrigues et al 2020 [64], Shelima et al 2007 [65], Fsahaye et al 2018 [66], Herago et al 2015 [67], Tanga et al 2019 [68], Upjohn et al 2013 [69], Upjohn et al 2012 [70], Usman et al 2015 [71], Biswas et al 2013 [72], Burn et al 2010 [24], Dennison et al 2007 [73], Popescu et al 2014a [74], Popescu et al 2011 [75], Popescu et al 2017 [76], Popescu et al 2012 [77], Pritchard et al 2005 [17].

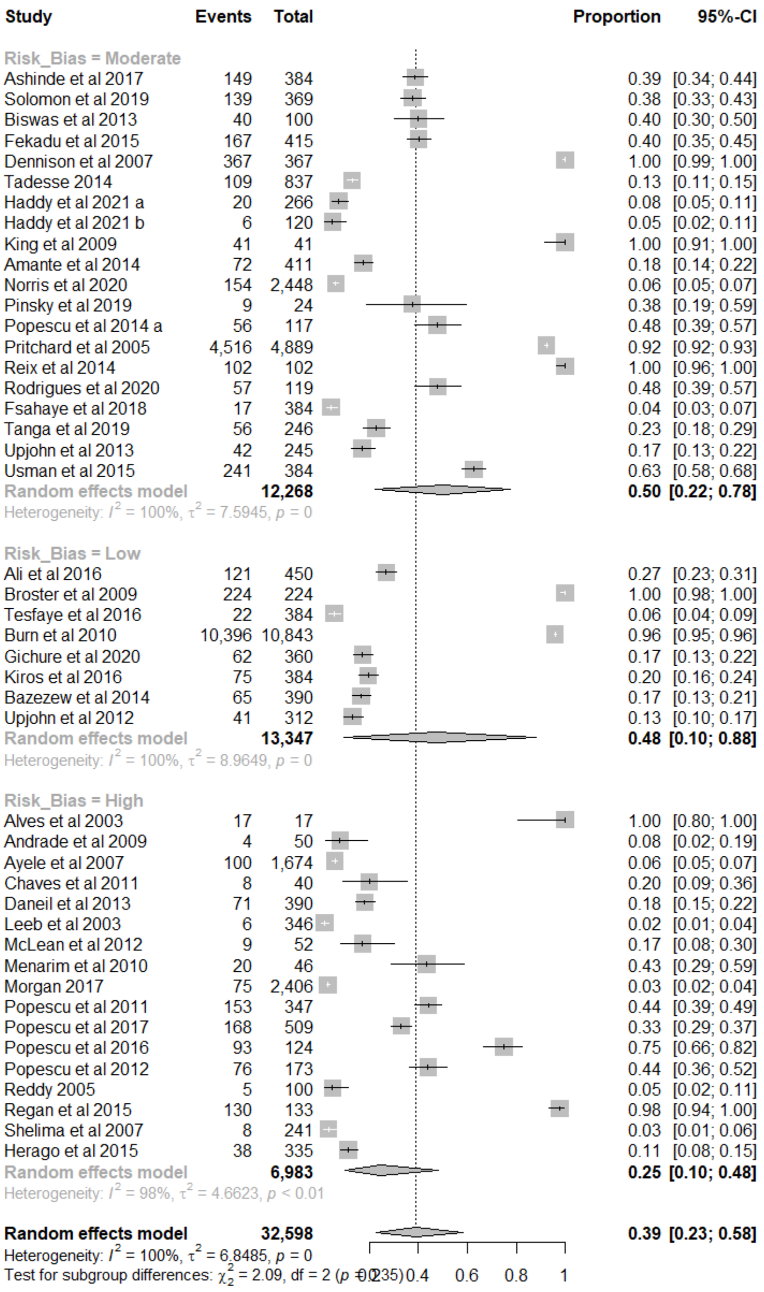

Supplement: Supplementary file 1 [file animals-12-03100-s001.zip › animals-1956041-New supplementary materials/Figure_S1-S5_Alternative_subgroup_forest_plots.pdf]
