# Supplementary material for: Prevalence and Factors Associated with Working Equid Lameness in Low- and Middle-Income Countries: A Systematic Review and Meta-Analysis
Source: Animals (Basel). 2022 Nov 10;12(22):3100. doi: 10.3390/ani12223100 (PMC9686919; doi:10.3390/ani12223100)
Supplement: Supplementary file 1 [file animals-12-03100-s001.zip › animals-1956041-New supplementary materials/Table_S1_Sensitivity_analysis.pdf]

| | No. Studies | Pooled Prevalence and 95% CI | $I^2$ | $t^2$ | Q statistic Subgroup differences | Metaregression Test of Moderators |
|---|---|---|---|---|---|---|
| **Pooled prevalence of lameness** | 41 | 0.30 [0.172;0.48] | 98.5% | 5.69 | | |
| **Pooled prevalence of abnormal gait** | 12 | 0.629 [0.307;0.867] | 99.7% | 5.49 | | |
| **Pooled prevalence estimates and subgroup analysis of lameness-related outcomes*** | | | | | | |
| ***Lameness-related outcomes*** | 45 | 0.389 [0.226;0.582] | 99.6% | 6.85 | *p*=0.025 | *p=0.024* |
| Lameness prevalence | 37 | 0.298 [0.160;0.487] | | | | |
| Gait abnormality prevalence | 8 | 0.788 [0.402;0.954] | | | | |
| ***Country income-level*** | 45 | 0.389 [0.226;0.582] | 99.6% | 6.85 | *p*=0.715 | *p=0.723* |
| Low-income | 26 | 0.353 [0.146;0.637] | | | | |
| Lower-middle income | 8 | 0.538 [0.199;0.845] | | | | |
| Upper-middle income | 11 | 0.369 [0.162;0.638] | | | | |
| ***Equid species*** | 45 | 0.389 [0.226;0.582] | 99.6% | 6.85 | *p*=0.213 | *p=0.913* |
| Horse | 14 | 0.453 [0.175;0.764] | | | | |
| Donkey | 17 | 0.305 [0.109;0.610] | | | | |
| Mule | 2 | 0.215 [0.152;0.294] | | | | |
| Pony | 1 | 0.375 [0.208;0.578] | | | | |
| Multiple species | 11 | 0.494 [0.140;0.854] | | | | |
| ***Gait assessed*** | 45 | 0.389 [0.226;0.582] | 99.6% | 6.85 | *p*=0.226 | *p=0.173* |
| Walk | 14 | 0.692 [0.247;0.939] | | | | |
| Walk and Trot | 4 | 0.232 [0.058;0.600] | | | | |
| Unknown | 27 | 0.285 [0.147;0.480] | | | | |
| ***Study risk of bias*** | 45 | 0.389 [0.226;0.582] | 99.6% | 6.85 | *p*=0.353 | *p=0.428* |
| Low | 8 | 0.475 [0.099;0.882] | | | | |
| Moderate | 20 | 0.500 [0.225;0.775] | | | | |
| High | 17 | 0.245 [0.102;0.480] | | | | |

## *Sensitivity Analysis*

| **Pooled prevalence estimates and subgroup analysis of lameness-related outcomes*** | | | | | Q statistic Subgroup differences | Excluding outlier with highest influence | Excluding high bias studies |
|---|---|---|---|---|---|---|---|
| ***Lameness-related outcomes*** | 45 | 0.389 [0.226;0.582] | 99.6% | 6.85 | *p*=0.025 | 0.37 [0.21;0.56] *p* =0.068 | 0.49 [0.25; 0.74] *p* =0.0280 |
| Lameness prevalence | 37 | 0.298 [0.160;0.487] | | | | 0.30 [0.16;0.49] | 0.35 [0.16;0.60] |
| Gait abnormality prevalence | 8 | 0.788 [0.402;0.954] | | | | 0.74 [0.30;0.95] | 0.92 [0.48;0.99] |
| ***Country income-level*** † | 43 | 0.351 [0.20;0.541] | 98.1% | 6.49 | *p*=0.562 | 0.351 [0.20;0.541] *p*=0.562 | 0.51 [0.25;0.76] *p* =0.2747 |
| Low-income | 24 | 0.287 [0.110;0.566] | | | | 0.29 [0.11;0.57] | 0.60 [0.24;0.88] |
| Lower-middle income | 8 | 0.538 [0.199;0.845] | | | | 0.54 [0.20;0.85] | 0.28 [0.17; 0.43] |
| Upper-middle income | 11 | 0.369 [0.162;0.638] | | | | 0.37 [0.16;0.64] | 0.49 [0.03;0.97] |
| ***Equid species*** | 31 | 0.369 [0.186;0.600] | 97.9% | 6.80 | *p*=0.50 | 0.38 [0.19;0.62] *p* =0.427 | 0.51 [0.17;0.84] *p* =0.2774 |
| Horse | 14 | 0.453 [0.175;0.764] | | | | 0.49 [0.18;0.81] | 0.81 [0.10; 0.99] |
| Donkey | 17 | 0.305 [0.109;0.610] | | | | 0.31 [0.11;0.61] | 0.32 [0.10;0.68] |
| ***Gait assessed*** | 18 | 0.573 [0.23; 0.86] | 99.5% | 9.95 | *p*=0.117 | 0.58 [0.22;0.88] *p* =0.1184 | 0.71 [0.23;0.95] *p* =0.0774 |
| Walk | 14 | 0.692 [0.247;0.939] | | | | 0.72 [0.23;0.95] | 0.76 [0.23;0.97] |
| Walk and Trot | 4 | 0.232 [0.058;0.600] | | | | 0.23 [0.06;0.60] | 0.27 [0.23;0.31] |
| ***Study risk of bias*** | 28 | 0.493 [0.25;0.74] | 99.7% | 8.01 | *p*=0.937 | 0.51 [0.25;0.76] *p* =0.9159 | |
| Low | 8 | 0.475 [0.099; 0.88] | | | | 0.54 [0.09;0.93] | |
| Moderate | 20 | 0.500 [0.225 0.775] | | | | 0.50 [0.22;0.76] | |

* Combines overall prevalence of lameness or gait abnormality reported by studies. Each study contributes as a single event. Studies reporting on prevalence of both lameness and gait abnormality were excluded from this analysis.

† Excludes multi-country studies with different income levels (n=2).
